# Chloroplast Transformation of *Platymonas* (*Tetraselmis*) *subcordiformis* with the *bar* Gene as Selectable Marker

**DOI:** 10.1371/journal.pone.0098607

**Published:** 2014-06-09

**Authors:** Yulin Cui, Song Qin, Peng Jiang

**Affiliations:** 1 Key Laboratory of Coastal Biology and Biological Resource Utilization, Yantai Institute of Coastal Zone Research, Chinese Academy of Sciences, Yantai, Shandong, China; 2 Key Laboratory of Experimental Marine Biology, Institute of Oceanology, Chinese Academy of Sciences, Qingdao, China; Arizona State University, United States of America

## Abstract

The objective of this research was to establish a chloroplast transformation technique for *Platymonas* (*Tetraselmis*) *subcordiformis*. Employing the *gfp* gene as a reporter and the *bar* gene as a selectable marker, transformation vectors of *P*. *subcordiformis* chloroplast were constructed with endogenous fragments *rrn16S*–*trnI* (left) and *trnA*–*rrn23S* (right) as a recombination site of the chloroplast genome. The plasmids were transferred into *P*. *subcordiformis* via particle bombardment. Confocal laser scanning microscopy indicated that the green fluorescence protein was localized in the chloroplast of *P. subcordiformis*, confirming the activity of the *Chlamydomonas reinhardtii* promoter. Cells transformed with the *bar* gene were selected using the herbicide Basta. Resistant colonies were analyzed by PCR and Southern blotting, and the results indicated that the *bar* gene was successfully integrated into the chloroplast genome via homologous recombination. The technique will improve genetic engineering of this alga.

## Introduction

*Platymonas* (*Tetraselmis*) *subcordiformis* is a marine unicellular green alga that is a widely used feed in aquaculture for its high nutrient levels. Recently, *P*. *subcordiformis* was observed to evolve H_2_ in a two-stage bio-photosynthesis, making it a potential resource for H_2_ [1]. In microalgae, photosynthetic hydrogen is catalyzed by hydrogenase with electric flow from electron transport chain of PSII in the chloroplast, while the activity of hydrogenase was inhibited by molecular oxygen evaluated from PSI.

Chloroplast transformation has many advantages over nuclear transformation, such as the high expression level of foreign genes [2], the homologous integration of foreign genes [3], the lack of gene silencing and position effects [4], and co-expression of multi-genes [5]. For *P. subcordiformis*, chloroplast transformation could be more suitable than nuclear transformation to raise its nutrient value. Meanwhile, a strategy to increase hydrogen production is to regulate PS I and PS II to maintain cells anaerobic by manipulating genes encoding PS proteins via chloroplast transformation. However, the current lack of chloroplast transformation protocols is obstructing this promising and fundamental research.

The first chloroplast transformation was reported in the green microalga *Chlamydomonas reinhardtii* [6]–[7]. The technology was rapidly applied to tobacco [8] and subsequently to many higher plant species [9]–[11]. However, algal plastid transformation research focused on *C. reinhardtii*, and only two other species [12]–[13] have stable chloroplast transformation systems.

An efficient selectable marker gene is critical for successful transformation. For chloroplast transformation, the commonly used primary markers are the *aadA* gene [14]–[15] and the *neo* gene [16], which confer resistance to spectinomycin–streptomycin and kanamycin, respectively; these antibiotics inhibit protein synthesis by the plastid ribosome [17]. We previously found [18] that *P. subcordiformis* was not sensitive to spectinomycin, streptomycin, or kanamycin but was very sensitive to the herbicide Basta. Basta contains a tripeptide of two Ala residues and an analog of Glu called phosphinothricin (PPT). After the two Ala are removed by an intracellular peptidase, the released PPT inhibits glutamine synthetase (GS) activity, leading to rapid buildup of intracellular ammonia, disruption of chloroplast structure, and cell death [19]. The *bar* gene encoding phosphinothricin acetyltransferase (PAT) confers tolerance to PPT. The interaction of PPT, GS, and PAT can be used to select positive colonies in nuclear transformations of higher plants and algae [18], [20]–[23]. In tobacco chloroplast transformation, the foreign *bar* gene could confer resistance to PPT in transformed plants but failed in direct selection for positive colonies [24]. This result may be related to the subcellular localizations of the expressed foreign PAT and GS isoforms.

In eukaryotic autotrophic cells, there are basically two isoforms of GS: GS1 localized in the cytoplasm and GS2 in chloroplasts and mitochondria [25]–[26]. In higher plants, the GS2 protein is usually encoded by a single nuclear gene and is expressed specifically in leaves [27]. The major role of GS2 is to re-assimilate the ammonium released by photorespiration in chloroplast and mitochondria.

In nuclear transformation, PAT localized in the cytoplasm could stop the PPT inhibition of GS1, which is critical to cell ammonia assimilation. The amount of GS1 is higher than that of GS2 in leaf cells. As a result, the recovered GS1 could then assimilate ammonia from photorespiration in chloroplasts before ammonia builds up to a level that disrupts chloroplast structure.

Unlike the single nuclear genome, plant cells contain multiple chloroplasts, each with multiple genomic copies. After transformation, there is usually one chloroplast with the foreign gene inserted into one copy of the genome. In the beginning of selection, the expressed level of PAT is much lower to detoxify PPT. The relatively low level of GS2 in one chloroplast compared with GS1 could not detoxify the inhibition of PPT to GS1 in cytoplasm in the time in order to the high level of ammonia, leading to cell death.

The subcellular localization of PAT, the amount of GS isoforms, and the lethal effect of PPT are the main factors for the failure of PPT in direct selection of tobacco chloroplast transformation. As a unicellular alga, transformed *P. subcordiformis* does not need the process of tissue culture without mature chloroplasts. Also, this alga has a single huge chloroplast, which may reduce the impact of PAT subcellular localization. Here, to understand the interaction between PAT and GS isoforms in algal chloroplasts, the *bar* gene was adopted as a selectable marker in *P. subcordiformis* chloroplast transformation.

## Materials and Methods

### Strain, Growth Condition, and Medium

*Platymonas subcordiformis* was obtained from Dalian Institute of Chemical and Physics, Chinese Academy of sciences, China. It was cultured in f/2 liquid medium or on agar plates at 23°C under a light intensity of 80–90 µmol photons m^−2 ^s^−1^, with a photoperiod of 12 h/12 h (light/darkness).

### Construction of Plasmids

The reported sequences of *P. subcordiformis* chloroplast genome were limited. Therefore, the chloroplast genomes of ten other green algae (Table 1) were used to design degenerate primers (Table 2) to isolate the fragment *rrn16* (16S rRNA)–*rrn23* (23S rRNA) in *P. subcordiformis*. Then, based on the sequence of *rrn16–rrn23*, the target fragments *rrn16*–*trnI* and *trnA*–*rrn23* were amplified by specific primers as described in table 3.

**Table 1:** Chloroplast genome of 10 green algae for degenerate primers.

Algae	GenBank accession number of chloroplast genome
*Chlamydomonas reinhardtii*	FJ423446
*Chlorella vulgaris*	NC_001865
*Dunaliella salina*	GQ250046
*Mesostigma viride*	NC_002186
*Nephroselmis olivacea*	NC_000927
*Oltmannsiellopsis viridis*	NC_008099
*Oocystis solitaria*	FJ968739
*Parachlorella kessleri*	FJ968741
*Pycnococcus provasolii*	NC_012097
*Scenedesmus obliquus*	DQ396875

**Table 2:** Degenerate primers for homologous region.

Primers	Sequences
16S/*trnA*-for	CACTGGGACTGAGACACG
16S/*trnA*-rev	CCSBYGRCVYCYGCMWTGC
*trnI*/23S-for	CAGYTGGTAGAGCRYYGCMYTT
*trnI*/23S-rev	CTTCGGCAGRYYDYTTAG
23S-for	CCCTTBAAAGAGTGCGTAA
23S-rev	AKTTTGCCGAGTTCCTTA

**Table 3:** Primers and restriction sites of the fragments for the chloroplast vectors.

fragments	Primers	Sequences	Enzymic sites	Length
*rrn16*–*trnI*	16-I for	cggggtaccTCCTACGGGAGGCAGCAGT	*KpnI*	1288 bp
	16-I rev	gcgtcgacTTGAGGCAAATGGGCTATGC	*SalI*	
*trnA*–*rrn23*	A-23 for	ttgcggccgcACAACGGAGTTTCGGAATA	*NotI*	1405 bp
	A-23 rev	cgagctcCGTTACTCAAACCGACATTC	*SacI*	
*atp*A 5′ UTR	5′*atp*A for	gcgtcgacAAGCTTATCGATGACTTTATTAG	*SalI*	668 bp
	5′*atp*A rev	ccgatatcGGACATTTTCACTTCTGGAGT	*EcoRV*	
*rbc*L 3′ UTR	3′*rbc*L for	cgggatccGTACTCAAGCTCGTAACGAAG	*BamHI*	448 bp
	3′*rbc*L rev	gctctagaGGATCGCACTCTACCGATT	*XbaI*	
the *bar* gene	bar for	ccgatatcATGAGCCCAGAACGACGCC	*EcoRV*	567 bp
	bar rev	cgggattcTCATCAAATCTCGGTGACGG	*BamHI*	
the *gfp* gene	GFP for	ccgatatcGTACTCAAGCTCGTAACGAAG	*EcoRV*	732 bp
	GFP rev	cgggattcGGATCGCACTCTACCGATT	*BamHI*	

Because of a lack of information on gene expression regulation in *P. subcordiformis* chloroplasts, the foreign gene cassette was made by amplifying a 5′ untranslated region (UTR) of *atp*A and a 3′ UTR of *rbc*L from a *Chlamydomonas reinhardtii* chloroplast transformation plasmid (p64D, provided by Biotechnology Research Institute, CAAS). The two fragments showed high efficiency in *C. reinhardtii* chloroplast transformation [28]. Meanwhile the *gfp* (green fluorescent protein) gene and the *bar* gene, were cloned respectively from pEGFP-N1 (Clontech, Palo Alto, CA, USA) and pSVB [23]. A unique restriction site was added to each fragment. Primers for these fragments are listed in Table 3. All the PCR products were sequenced by Sangon Biotech (Shanghai, China).

The resulting fragments were then cloned into the pMD18-T vector individually with a TA cloning kit (Tiangen, China). After restriction enzyme digestion, the fragments were inserted into the plasmid pBluescript KS (+) (Promega, USA) in the fit sites one by one. The plasmid containing the *gfp* gene was named as pPSC-GFP, while that containing the *bar* gene was pPSCB.

### Microparticle Bombardment

The pPSC*–*GFP and pPSCB plasmids were used for *P. subcordiformis* chloroplast transformation. Alga preparation and bombardment parameters were as previously described [18]. Gold particles 0.8–1.5 µm in diameter were used as plasmid carriers, and 2–3 µg of plasmid DNA was used for each bombardment following protocols described by Bio-Rad (Hercules, CA, USA). The rupture pressure was 900 psi and the bombardment distance 6 cm. The cells bombarded with uncoated gold particles were set as negative controls. All experiments were performed in triplicated.

### Microscopy Analysis

Cells transformed with pPSC*–*GFP were first examined with a Nikon Eclipse 50i microscope (Nikon, Tokyo, Japan) with blue-light excitation 48 h after bombardment. Transformed cells were selected by comparing their fluorescence intensity with that of negative controls using a fluorescence-assisted cell sorting (FACS) (Vantage SE, Becton Dickinson, Franklin Lakes, NJ, USA) with 488 nm excitation and standard filter set-up. Then, the localization of GFP in the harvested cells was examined by confocal laser scanning microscope (CLSM) (FluoView FV1000, Olympus, Tokyo, Japan) with excitation at 488 nm and 635 nm for GFP and chlorophyll fluorescence, respectively.

### Herbicide Selection

After 2 days of recovery, the cells transformed with pPSCB were transferred to selective f/2 medium with 15 µg ml^−1^ of Basta for a month. Then, surviving cells were spread on solid medium with 10 µg ml^−1^ of Basta. Colonies appearing after 2 weeks were selected and streaked on agar plates of f/2 medium with 5 µg ml^−1^ Basta. After another 2 weeks of cultivation, 40 colonies were selected and cultured in liquid f/2 medium without the herbicide.

### Detection of the Integration Events

**Figure 1:**
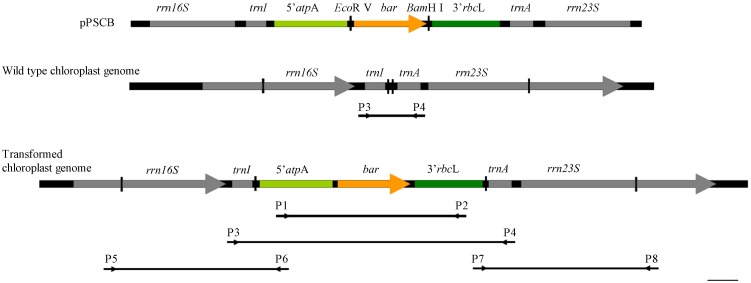
Construct map of homologous recombinant in *P*. *subcordiformis* chloroplast. Bar = 100 bp.

After 3 weeks of cultivation, cells originating from resistant colonies were harvested and genomic DNA was extracted using a plant genomic DNA kit (Tiangen). According to the map of pPSCB and the construct of the *P. subcordiformis* chloroplast genome (Fig. 1), four pairs of primers (Table 4) were designed to examine the *bar* gene in these resistant colonies. A fragment of 5′*atp*A–bar–3′*rbc*L was amplified from genomic DNA using primers p1 and p2 to confirm the existence of the *bar* gene. Colonies with positive amplification were further analyzed by PCR with primers p5 and p6, p7 and p8 to test homologous integration, and with primers p3 and p4 to test for homoplasmic integration. All PCR products were sequenced.

**Table 4:** Primers for testing homologous recombination in *P*. *subcordiformis* chloroplast.

Primers	Sequences
P1	GCTTATCGATGACTTTATTAG
P2	GGATCGCACTCTACCGATT
P3	ACAAGCAACGGGCTATTA
P4	TTTAGGCTGTTCCCATTT
P5	TATGCTGAGGAGTAAAACGGTA
P6	ACAGGCGTCGTAAGCAACTA
P7	AAGCGGATGTAACTCAAT
P8	TCCTGACTAACCCTCCAT

Finally, samples with positive amplification in all PCR steps were analyzed by Southern blotting. A fragment of pSVB amplified with primers a (5′-GCACCATCGTCAACCACTA-3′) and b (5′-CAGAAACCCACGTCATGC-3′) (Tan et al. 2005) was used as the probe. Total DNA (≥4 µg) of each sample was first digested with *Eco*R V and *Bam*H I. Southern blotting was conducted with a DIG DNA labeling and detection kit (Roche, Penzberg, Germany).

## Results

### Plasmids

The plastid fragment cloned from *P. subcordiformis* was 3729 bp in length (GenBank no. JN561782) with the organization *rrn16*–*trnI–trnA*–*rrn23*, which is similar to that of higher plants. Fragments of *rrn16*–*trnI* (1288 bp) and *trnA*–*rrn23* (1405 bp) were separated as the homologues regions for plastid transformation vectors.

**Figure 2:**
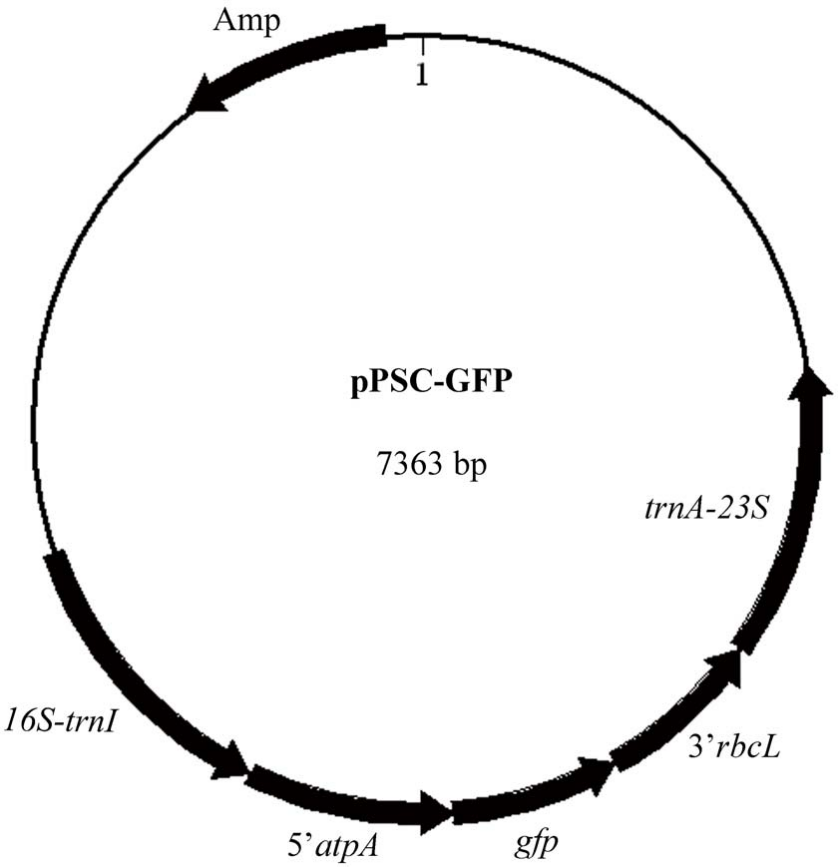
Physical map of the vectors pPSC-GFP.

Two vectors, pPSCB (KJ668650) and pPSC*–*GFP (KJ668651), for *P. subcordiformis* chloroplast transformation were constructed. Maps of the two plasmids were similar, as described in Fig. 2. In the plasmid pPSCB, the *bar* gene was used as the selectable marker to confer algal resistance to the herbicide Basta, while the plasmid pPSC*–*GFP used the *gfp* gene as the reporter. The *bar* and *gfp* genes were each driven by the *C. reinhardtii* chloroplast regulators 5′ UTR of *atp*A and 3′ UTR of *rbc*L. The expression cassettes of the *bar* and *gfp* genes in the respective plasmids were both between the plastid border regions *rrn16*–*trnI* and *trnA*–*rrn23*, so homologous recombination of the foreign genes was allowed.

### GFP Expression

**Figure 3:**
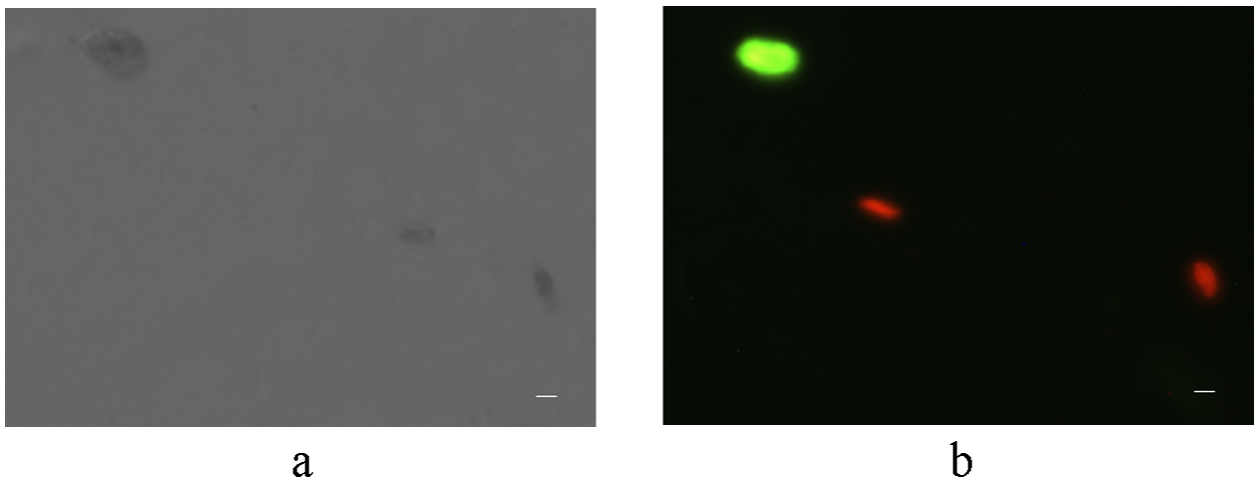
GFP fluorescence of Transformed *P. subcordiformis* by Microscope. Fig. 3a, View of cells under white light; Fig. 3b, View of cells under fluorescence excitation. Bar = 5 µm.

After bombardment of pPSC*–*GFP, some cells showed green*–*yellow fluorescence with blue-light excitation under fluorescence microscope, while the negative controls showed red chlorophyll fluorescence (Fig. 3). This result indicated that GFP was expressed in some cells.

**Figure 4:**
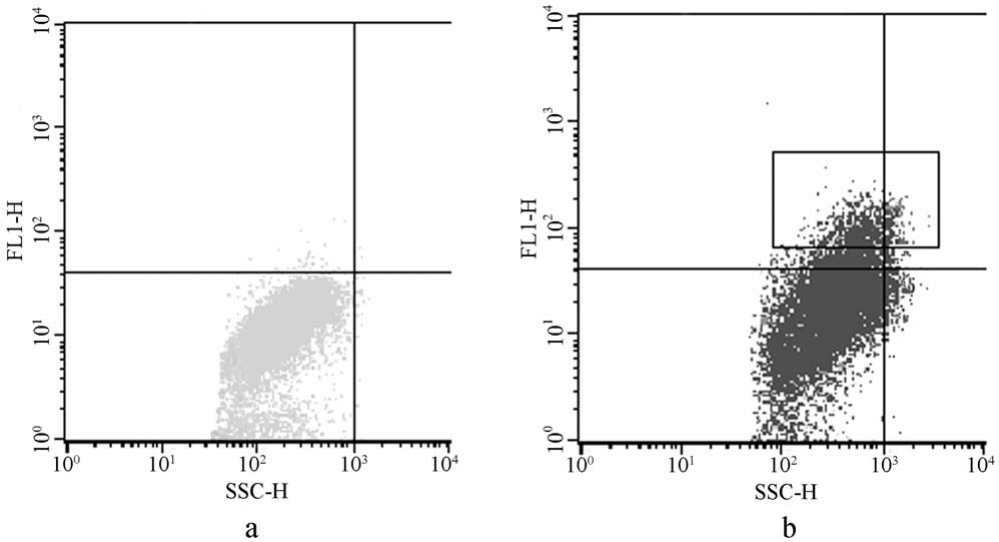
Quantitative comparison fluorescence variant between cells of the negative controls and the transformed lines. The axis FL1-H means relative intensity of GFP florescence, and the axis SSC-H means side scatter of each alga cell. Fig. 4a, Fluorescence intensity of the negative cells. Fig. 4b, Fluorescence intensity of the transformed cells. The cells with higher fluoresce intension in the rectangle in Fig. 4b, were harvested for further LSCM scanning for GFP positive cells. These cells may contain wild type cells, cells with plasmids inserted into chromosomes, and cells with plasmids inserted in chloroplast genome.

Chlorophyll fluorescences with an excitation wavelength of 488 nm, but the cells transformed with GFP showed more fluorescence with 488 nm excitation than did the negative controls. Therefore, we quantitatively compared these fluorescence differences by FACS, and the cells with more intense fluorescence (box in Fig. 4b) were harvested for further CLSM analysis. The positive fraction was 4.95% in the transformed cells.

**Figure 5:**
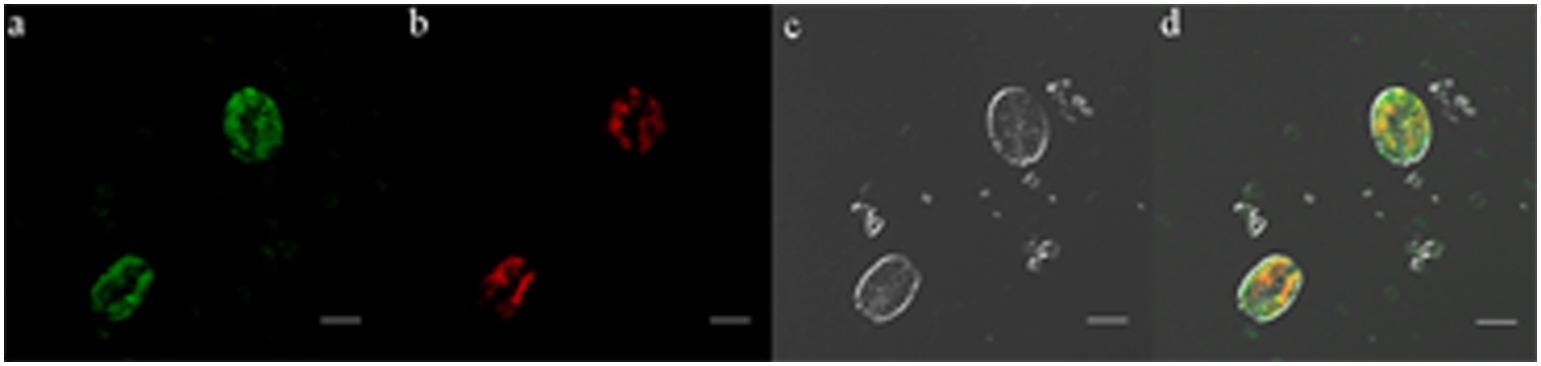
Imagines of GFP and chlorophyll fluorescence in *P. subcordiformis* by LSCM. Fig. 5a, GFP fluorescence. Fig. 5b, Chlorophyll fluorescence. Fig. 5c, Imagine with white light. Fig. 5d, Combination of the imagines a, b and c. Bar = 5 µm.

By combining images of GFP and chlorophyll fluorescence in some harvested cells by CLSM scanning (Fig. 5), GFP was found to be co-localized with chlorophyll in the cup-shaped chloroplasts of *P. subcordiformis*. The GFP fluorescence in *P. subcordiformis* chloroplasts indicated the activity of the regulating factors from *C. reinhardtii* in *P. subcordiformis*. The positive rate of pPSC-GFP transformed to *P. subcordiformis* chloroplast is 500–1000 cell per 1 µg DNA according to fifteen independent experiments.

### Detection of the *Bar* Gene Integration

**Figure 6:**
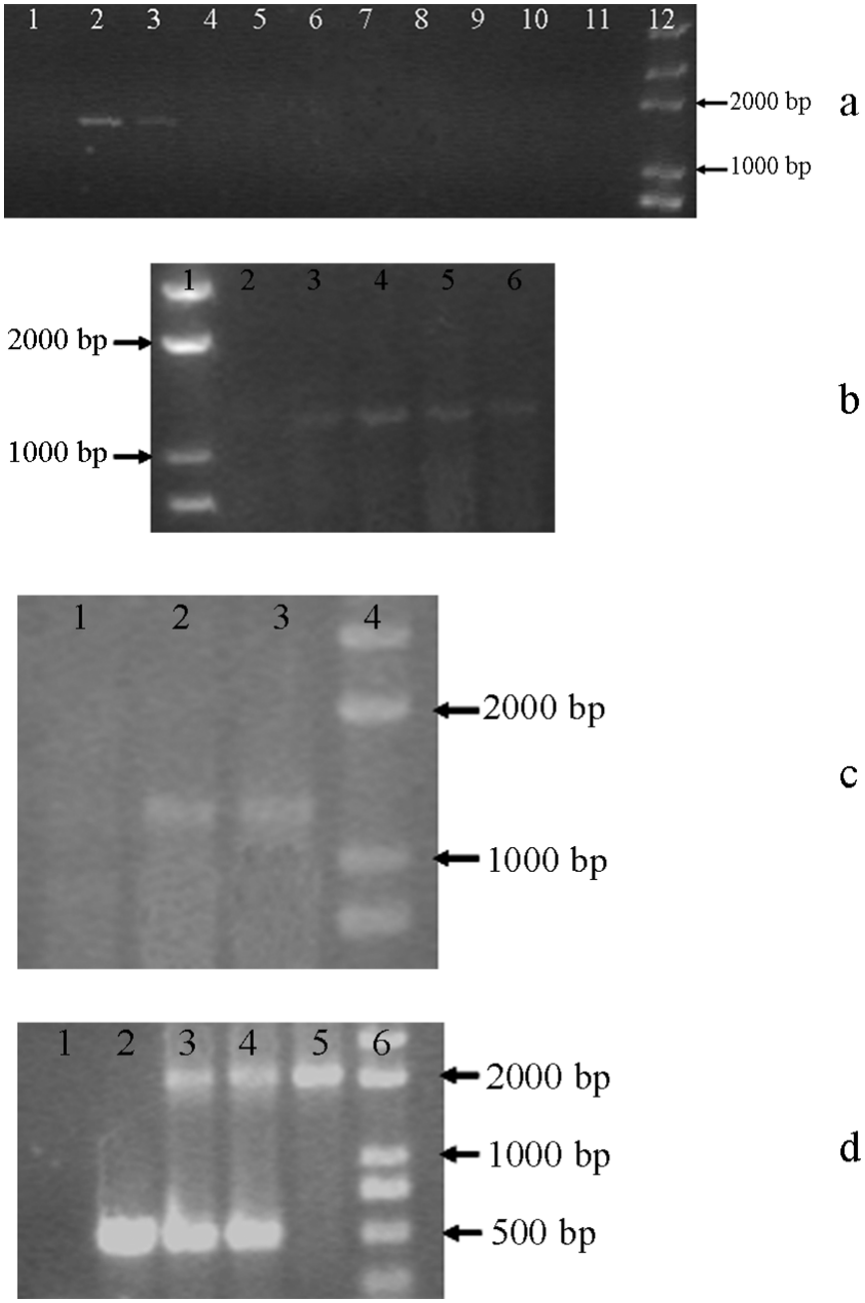
PCR detection of resistant *P. subcordiformis* colonies. Fig. 6a, PCR product with primers p1 and p2. Lane 1, wild type of *P*. *subcordiformis*; lane 2, PSB1; lane3, PSB2; lanes 4–11, other resistant colonies; lane 12, DNA Marker Trans 2K. Fig. 6b, PCR product with primers p5 and p6. Lane 1, DNA Marker Trans 2K; lane 2, wild type of *P*. *subcordiformis*; lanes 3–6, resistant colonies. Fig. 6c, PCR product with primers p7 and p8. Lane 1, wild type of *P*. *subcordiformis*; lanes 2 and 3, PSB1 and PSB2; lane 4, DNA Marker Trans 2K. Fig. 6d is PCR product with primers p3 and p4. Lane 1, negative control; lane 2, wild type of *P*. *subcordiformis*; lanes 3 and 4, PSB1 and PSB2; lane 5, plasmid pPSCB; lane 6, DNA Marker Trans 2K.

**Figure 7:**
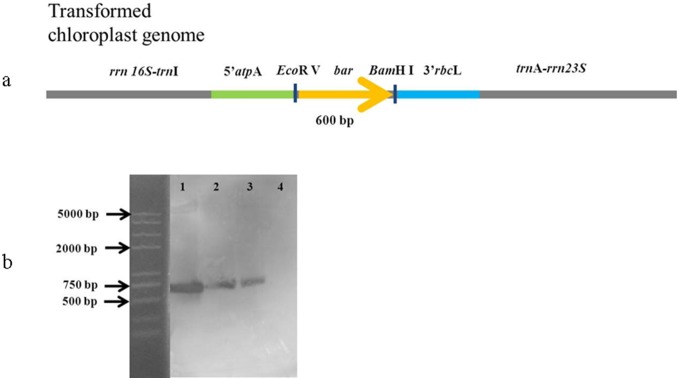
Southern blotting analysis of the PCR-positive *P. subcordiformis* colonies. Total DNA (≥4 µg) of each sample was firstly digested with *Eco*R V and *Bam*H I. The probe was hybridized with the *bar* gene. The size of hybridized fragment should be 600 bp. Lane 1, pPSCB; lane 2, PSCB1; Lane 3, PSCB2; lane 4, wild-type of *P*. *subcordiformis*.

Herbicide resistance was tested first by cultivating the bombarded alga in f/2 medium containing Basta. After selection, 40 resistant colonies were chosen for further PCR detection. Two colonies (named PSB1 and PSB2) gave positive results with all four pairs of primers (Fig. 6). The bands of about 1700 bp with the correct sequence (Fig. 6a) indicated the presence of the *bar* gene expression cassette in *P. subcordiformis*. The bands of about 1300 bp (Fig. 6b) indicated the homologous insertion in the left border, while the bands of 1500 bp (Fig. 6c) indicated the homologous insertion in the right border. Primers p3 and p4 could yield three types results: a single band of about 500 bp, which represents the wild type colony; a single band of about 2000 bp, which represents the homoplasmic colony, or two bands of 500 bp and 2000 bp, representing a non-homoplasmic transformed colony. Both colonies produced two bands with these primers (Fig. 6d), indicating that the *bar* gene has been inserted into some chloroplast genomes in each. Homoplasmic strains can be obtained by repeated selection. The green alga *C. reinhardtii* [29] and the red alga *Porphyridium* [13] yielded homoplasmic mutants after more than 6 month selection. Ultimately, both colonies showed positive bands in the Southern blotting analysis (Fig. 7), demonstrating that the *bar* gene was stably integrated into the *P*. *subcordiformis* chloroplast genome.

## Discussion

Here, a stable transformation of *P. subcordiformis* chloroplast was achieved using the *gfp* gene as a reporter and the *bar* gene as a selectable marker. This technique will facilitate the genetic manipulation of this organism to express foreign genes or modify the expression of endogenous ones. This successful algal chloroplast transformation system will promote research on other algal chloroplast systems.

An efficient chloroplast transformation system requires four parts, including an endogenous plastome fragment as a homologous insertion site, a promoter originating from endogenous or heterogeneous chloroplasts, a suitable selectable marker or a reporter gene, and a practical method to transfer the foreign plasmid into host cells. Particle bombardment is the most widely used method for plasmid delivery into chloroplasts [6], [8] and the transforming frequency is quite high. In the present research, particle bombardment was successfully used to transfer the foreign plasmid into the *P. subcordiformis* chloroplast. However, this method was less efficient for nuclear transformation of *P. subcordiformis* than glass-bead agitation [18]. This difference may be related to the fact that *P. subcordiformis* has a single large cup-shaped chloroplast surrounding the nucleolus, which may facilitate particles entering the former while blocking them from the latter.

The *gfp* gene is a widely-used reporter gene for chloroplast transformation [30]–[32]. In photosynthetic cells, chlorophyll and heterogonous GFP can fluoresce with excitation. Based on the difference in fluorescence between chlorophyll and GFP, positively-transformed cells were harvested by FACS [33]–[34]. The difference was also used to obtain separate fluorescence images of both chlorophyll and GFP by CLSM [30], [35], allowing GFP to be localized in the chloroplast.

*Platymonas subcordiformis* is compressed and obovate in outline with dimensions of 11*–*16 µm in length, 7*–*9 µm in breadth and 3.5*–*5 µm in depth [36]. Under CLSM, the cells always lay flat, with depth as the Z-axis, which resulted in just five to seven images during Z-series scanning. These images were not sufficient for a very clear three-dimensional image. Further research is needed to find a method to orient *P. subcordiformis* cells so that the length is the Z-axis, and technical improvements are also needed for a thinner optical section to obtain more images within a Z-axis to create a clear three-dimensional image.

The *bar* gene conferring resistance to Basta is an excellent selective marker in nuclear transformation of plants [20]–[21] and algae [18], [22]–[23], but it failed in the direct selection of tobacco chloroplast transformation [24]. The reasons for this failure are not presently clear and predicted from the lethal selection of Basta in the initial selection of the tissue culture before a sufficient proportion of the homologous recombinant plastomes established, while the selection of antibiotics was non-lethal [37].

In the present research, we tested the *bar* gene as the direct selectable marker and found that *bar* is suitable for *P. subcordiformis* chloroplast transformation. The results may be explained in two ways. First, as a unicellular alga with rapid growth rate, the transformed *P. subcordiformis* could recover within about 8 h of dark culture, unlike the long tissue culture period in tobacco leaf transformation. Also, because the alga has a single chloroplast, the plastome inserted with foreign gene could be easier to reach a high portion in the chloroplast. As a result, the *bar* gene could be expressed quickly and at high levels in *P. subcordiformis*. This is the first research to employ the *bar* gene as a selectable marker in algal plastid transformation. Further research is needed on expression pattern of the *bar* gene in the *P. subcordiformis* chloroplast.

## Conclusion

This paper reports the successful chloroplast transformation of *P. subcordiformis*. Much further work is needed to develop a sophisticated system. The regulation of gene expression in *P. subcordiformis* chloroplasts is in urgent need for the endogenous promoters. Furthermore, to express several genes at the same time, the construct of a polycistron in this alga is needed.
